# Effects of In Ovo Injection of α-Ketoglutaric Acid on Hatchability, Growth, Plasma Metabolites, and Antioxidant Status of Broilers

**DOI:** 10.3390/antiox11112102

**Published:** 2022-10-25

**Authors:** Vaishali Gupta, Chris Major Ncho, Akshat Goel, Chae-Mi Jeong, Yang-Ho Choi

**Affiliations:** 1 Department of Animal Science, Gyeongsang National University, Jinju 52828, Korea; 2 Division of Applied Life Sciences (BK21 Plus Program), Gyeongsang National University, Jinju 52828, Korea; 3 Institute of Agriculture and Life Sciences, Gyeongsang National University, Jinju 52828, Korea

**Keywords:** AKG, anti-oxidative capacity, broilers, in ovo feeding, gene expression

## Abstract

Recently, α-ketoglutaric acid (AKG) has gained importance as an antioxidant. Its dietary supplementation in animals and humans has proved beneficial. Moreover, an extensive group of studies on in ovo feeding has proved that it produces better day-old chicks and overall performance. Combining the two, we hypothesized that in ovo feeding of AKG could improve the antioxidant status in addition to chick quality and broiler performance. At 17.5 days of incubation, eggs were divided into one of five groups: eggs that received (i) no injection (U-CON), (ii) distilled water (DDW) only (0 AKG), (iii) 0.5% AKG dissolved in DDW (0.5 AKG), (iv) 1.0% AKG dissolved in DDW (1.0 AKG), or (v) 1.5% AKG dissolved in DDW (1.5 AKG). Chicks were raised until 21 days of age. Biological samples were collected on day 0 and day 21. Body weight (*p* = 0.020), average daily gain (*p* = 0.025), and average daily feed intake (*p* = 0.036) were found to quadratically increase with the amount of AKG during the grower phase. At day 0, the absolute (*p* = 0.040) and relative weight (*p* = 0.035) of the liver increased linearly with an increasing amount of AKG. The 0.5 AKG group had significantly higher plasma protein (*p* = 0.025), absolute and relative heart indices at day 0 (*p* = 0.006). An in ovo feeding of AKG improved the plasma antioxidant capacity of chicks at day 0 as compared to 0 AKG. AKG effect was seen on the plasma antioxidant balance, which increased linearly with the increasing dose of in ovo AKG. Furthermore, 1.0 AKG and 1.5 AKG showed a significant (*p* = 0.002) upregulation of the hepatic mRNA expression of nuclear factor erythroid 2-related factor (NRF2) in comparison to 0 AKG. The results imply that without negatively affecting hatchability performance, in ovo feeding of AKG has beneficial effects on the antioxidant status of broilers.

## 1. Introduction

Over the past 50 years, the broiler industry has seen significant improvements in feed-conversion rates, as well as an increase in broiler weight at marketable age [1]. Modern-day broilers have emerged as a sequel of continuous selection in the past several decades. They are genetically superior and perform the best under optimal management conditions [2]. However, their ability to resist harsh weather conditions is minimal when compared to slow-growing broilers [3]. Due to the global rise in temperature, birds suffer from heat stress [4]. Heat stress is also identified as one of the major progenitors of oxidative stress in birds [5]. Animals, including poultry, generate reactive oxygen species (ROS) and reactive nitrogen species (RNS) as part of their daily metabolic processes in the mitochondria of the cell [6]. RNS are oxidative and nitrosative molecules that trigger the degeneration of lipids and proteins in the pectoralis major muscle of broilers [7]. During heat stress, ROS production increases in the cells leading to increased oxidative stress [8]. Phospholipids are the constituents of cell membranes and are easily oxidized [9]. Oxidative stress leads to the disruption of tight junctions in the gut [10], facilitating the penetration of pathogens into the bloodstream through the enterocytes, rendering the birds more susceptible to diseases [11].

The development of chicken embryos completely relies on the nutrients present in the egg. Embryonic life contributes about 33% of the period from the start of incubation to the day when the broilers are marketed, which denotes the significance of embryonic life in the broiler industry. During the later stages of the incubation and hatching process, the oxygen utilization increases manyfold to meet the high metabolic rates [12], leading to higher production of ROS [13]. At this stage, the yolk lipids are highly susceptible to being oxidized by ROS [14]. Hence, this can cause the deterioration of biomolecules and membranes, which can adversely affect the development of embryos [15].

In ovo feeding targets the perinatal period of development [16]. In 1982, Sharma and Burmester introduced in ovo vaccination for Marek’s disease [17], which led to the emergence of the concept of in ovo feeding in the poultry industry. This has been explored as a method to provide extra nutrition to the growing embryo during incubation to produce better day-old chicks. In ovo feeding of L-arginine at day 18 of incubation, for example, resulted in reduced malondialdehyde (MDA) concentrations in the breast muscle of slow-growing broilers at hatching, increased the total antioxidant capacity (TAC) at 21 days of age, and improved meat quality at 63 days of age [18]. A recent finding in geese showed that in ovo feeding of methionine helped reduce the MDA concentrations and improved glutathione levels at hatching [19]. Furthermore, in ovo feeding of threonine led to better humoral immunity responses in broilers [20].

α-Ketoglutaric acid (AKG) has been identified as one of the most important speed determinants in the tricarboxylic acid cycle in a majority of organisms [21]. AKG can be synthesized when glucose or oxaloacetate is combined with pyruvate, giving it another synonym as 2-oxaloglutarate and 2-ketoglutarate [22]. Tissues convert AKG to glutamine, which acts as a source of energy for cells [23]. AKG is a precursor to glutamine and is readily soluble in water without showing toxic effects [21]. Dietary AKG supplementation has been shown to improve skeletal development in turkeys [24], intestinal energy levels and antioxidative capacity in ducks [25], and meat quality in laying hens [26]. AKG has been shown to reduce oxidative stress, activate the antioxidant system, and improve the resistance of S. cerevisiae cells to hydrogen peroxide [27]. In grass carps, AKG supplementation led to a significant increase in the glutathione peroxidase 1 (GPX1), catalase (CAT), and superoxide dismutase (SOD) activities, while reducing the malondialdehyde (MDA) concentration in blood [28]. Moreover, AKG administered to rats through the nasal route had beneficial effects in protecting the lung tissues from oxidative damage caused due to ammonia [29] probably by improving SOD, CAT, and glutathione (GSH) activities and reducing MDA levels and lactate dehydrogenase (LDH) activity. Hence, AKG supplementation has been proved useful in alleviating oxidative stress and/or improving the antioxidant defense mechanism in a range of organisms, such as single-cell organisms [27,30] rats [29,31,32,33], fishes [28], and poultry [25,26,29,34,35]. 

To our knowledge, there are no studies eliciting the prospective multitudinous effects of in ovo feeding of AKG in broilers. Therefore, this study was designed to probe the overall effects of in ovo feeding of AKG on plasma antioxidant status, hepatic antioxidant-related mRNA expression, hatchability parameters, production performance, organ index, and plasma metabolites in broilers at hatching and 21 days old.

## 2. Materials and Methods

The present experiment was conducted at the research facility of the Gyeongsang National University, Korea. All the experimental procedures were approved by the Institutional Animal Care and Use Committee of the Gyeongsang National University (GNU-200916-C0058).

### 2.1. Egg Incubation and In Ovo Feeding

A total of 870 eggs were obtained from a commercial local farm (Harim Hatchery, Iksan, South Korea). The age of the breeder hens was about 40 weeks old. The eggs were weighed (53.80 ± 0.11) and labeled individually before being transferred into the incubator (Rcom Co., Ltd., Kimhae, Korea). On day 10 of incubation, the eggs were candled, and the infertile eggs were removed. A second candling was performed on day 17 to remove any other remaining infertile eggs. Finally, 350 eggs were selected using the Solver module of Microsoft Excel (Microsoft Excel 2016; Microsoft Corp., Redmond, WA, USA) and distributed into one of 5 groups of equal numbers with similar weights. The procedure to prepare the solutions was followed as per discussed in a previous study [36]. Briefly, AKG (#75890, Sigma-Aldrich, St. Louis, MO, USA) was dissolved in distilled water (DDW) to prepare 0.5%, 1.0%, and 1.5% w/v solutions of AKG. The solutions were kept at a temperature of 30℃ until they were used. The treatment groups are as follows: (i) uninjected control (U-CON) (n = 70); (ii) 0%AKG-injected control (0 AKG) (n = 70); (iii) 0.5% AKG-injected group (0.5 AKG) (n = 70); (iv) 1.0% AKG-injected group (1.0 AKG) (n = 70); and (v) 1.5% AKG-injected group (1.5 AKG) (n = 70).

The protocol in ovo was exactly followed as discussed in our previous studies [37]. At 17.5 days of incubation, all the eggs except for the U-CON group received an injection of 0.6 mL of AKG solutions as per their groups. A small area towards the broad end of the egg was disinfected with 70% ethanol and a tiny hole using a dental drill (Saeshin, Daegu, Korea) was drilled, through which a syringe with a 1-inch long 23-G needle was inserted. The hole was sealed with surgical tape (3M Micropore, Saint Paul, MN, USA) after injection. The U-CON group, which did not receive any in ovo injection, was also removed from the incubator for a time similar to that which it took to perform the in ovo injection to neutralize the effect in all treatment groups. On the day of pulling the hatch, hatching parameters were recorded. The eggs which did not hatch were broken manually and their developmental stages were visually examined.

### 2.2. Rearing and Feeding of Birds

After recording the hatchability parameters, the U-CON treatment was removed and a total of 168 chicks (42 birds per treatment) were reared in battery cages (6 cages per treatment) where one cage containing 7 birds was considered as one replicate. The chicks were raised as per standard guidelines of temperature and relative humidity described for Arbor Acres broilers (Arbor Acres, Broiler Pocket Guide, 2018). Feed and water were provided ad libitum to the birds. The birds, reared for 21 days, were provided with commercial starter feed for the first week, followed by commercial grower feed for the next two weeks (Broiler Luxury, Nonghyup Feed, Gyeongju, South Korea). Body weight (BW) and feed intake (FI) were recorded for the birds on weekly basis. Feed consumption was calculated by subtracting the feed remaining in the feeders from the total amount of feed offered to the birds. The feed-conversion ratio (FCR) was calculated as the ratio of feed consumed by the birds to the body-weight gain (BWG) every week during the experimental period.

### 2.3. Sample Collection

Biological samples were collected twice, at hatching and 21 days of age. At hatching, one chick from each cage of each treatment (0 AKG, 0.5 AKG, 1.0 AKG, and 1.5 AKG) was randomly selected and sacrificed by severing the jugular vein of the birds. Hence, six birds per treatment were sampled. The blood collected was transferred into heparinized vacuum containers (#367874, BD Co., Ltd., Franklin Lakes, NJ, USA). Blood samples were then centrifuged at 2000× *g* for 10 min at 4 °C. Plasma was collected and stored at −20 °C for later analysis. Tissues (liver, yolk sac, heart, proventriculus, and gizzard) were cut free and weighed. The liver samples were collected, immediately snap-frozen in liquid nitrogen, and stored at −80 °C for later analysis. 

The second sample collection was performed at 21 days of age and the procedure followed was similar to our previous studies [38]. Six birds were randomly selected from each treatment group and euthanized in a chamber containing carbon dioxide. Blood was collected using sterile syringes and processed as previously discussed by Ncho et al. [36]. The weight of the liver, spleen, and bursa was measured and the lengths of different parts of the intestine (duodenum, jejunum, and ileum) and ceca were recorded. Hepatic samples were collected and stored similarly to the method described above.

### 2.4. Determination of Metabolite Concentrations and Antioxidant Capacity in Plasma

Calcium, cholesterol, glucose, total protein, and triglyceride concentrations in plasma were measured using a VetTest Chemistry Analyzer (IDEXX Co., Ltd., Westbrook, ME, USA) with dry-slide technology following the manufacturer’s guide. The total anti-oxidative capacity (TAC) was estimated by the method previously described by Gerasopoulos et al. [39]. In this method, 20 µL of plasma was diluted using a 480 µL solution of sodium-potassium phosphate (pH 7.4) after which 500 µL of 0.1 mmol/L of 2,2-diphenyl-1-picrylhydrazyl (DPPH) free radical was mixed in the diluted plasma samples. The resultant mixture was incubated at room temperature for 30 min in the dark. Subsequently, the samples were centrifuged at 10,000× *g* for 6 min and, finally, the absorbance was read at 517 nm. A control solution was prepared using 500 µL of 0.1 mmol/L of DPPH reagent and 480 µL of sodium-potassium phosphate. The inhibitory activity was expressed as a percentage using the formula:

% inhibition = [1 − (A1/A0)] × 100,

where A0 is the absorbance of the control and A1 is the absorbance of the test samples.

The protocol for measuring the concentration of MDA was followed as described by Jyothi et al. [40] with little modification. Briefly, in a tube, 400 µL plasma was added to an equal volume of 40% trichloroacetic acid (TCA) and 800 µL of 0.67% thiobarbituric acid (TBA) was added to the mixture. This mixture was thoroughly vortexed after which it was incubated in a water bath at 95 °C for 45 min. After cooling on ice for 5 min, the samples were centrifuged at 1000× *g* for 6 min. Lastly, the absorbance of their supernatants was read at 530 nm. The final concentration of MDA was estimated by the formula:

MDA concentration (mol/L) = A/(K × L),

where A is the absorbance of the sample, K is the molar extinction coefficient (1.5 × 10^5^), and L is the length of the cuvette used (1 cm).

The antioxidant balance (AB) was calculated as the ratio of DPPH-RSA value to the MDA value as previously described [37].

### 2.5. Real-Time PCR for mRNA Quantification

Trizol reagent (Catalogue# 15596018, Thermo Fisher Scientific, Waltham, MA, USA) was used to extract RNA from 50 mg of the liver tissue as per the manufacturer’s guide. The concentrations and purities of the samples were measured as the ratio of optical density at 260 and 280 nm using a Nanodrop (Thermo Scientific, Waltham, MA, USA). After confirming the purity of the RNA, a Verso cDNA Synthesis Kit (Catalogue# AB1453 A, Thermo Fisher Scientific) was used following the manufacturer’s guide to synthesize cDNA from the RNA samples. The cDNA thus obtained was stored at −20 °C and used for amplification of different genes with the help of a StepOnePlus real-time PCR system (Life Technologies, Carlsbad, CA, USA). A 10 µL Power SYBRTM green PCR master mix (Catalogue# 4312704, Life Technologies, Carlsbad, CA, USA), along with 10 pmol of forward and reverse primers specific for each gene and cDNA was included in each reaction. Table 1 represents the primer sequences of the genes used in the current study. The total volume of one reaction was made up to 20 µL. GAPDH and β actin were used as housekeeping genes. Target-gene quantification was normalized with the Ct values of GAPDH and β actin. Similar to the previous studies, the fold change (FC) was determined using the 2–ΔΔCt algorithm [41]. The values were presented as log2 FC to evaluate the regulation of the gene expression as was described before [42].

### 2.6. Statistical Analysis

An entire cage or replicate was considered as an experimental unit for all the evaluated response parameters in this study. Before using appropriate parametric tests, the normality of distribution and homoscedasticity assumptions were assessed using the Shapiro–Wilk and Levene’s tests, respectively. Parameters (plasma metabolites, plasma antioxidant capacity, and hepatic mRNA expression) recorded on day 0 and day 21 were analyzed via two-way ANOVA with the main effects as AKG concentration (0%, 0.5%, 1.0%, and 1.5% AKG) and age (Day 0 and Day 21). Other parameters such as hatchability and production parameters were analyzed using one-way ANOVA. Following a significant *p*-value (*p* < 0.05), a Tukey’s post hoc test was used to assess differences between means. In addition, dose-related effects of AKG were analyzed using polynomial regression analysis in the absence of the U-CON group.

For multivariate analysis, principle component analysis (PCA) was conducted with oxidative stress-related parameters (gene expression, DPPH-RSA, MDA, and AB) as primary variables. Metadata such as Day, AKG concentration, and treatments were considered supplementary variables used to highlight individuals in the PCA plots. Finally, as previously performed [45], a hierarchically clustered heatmap was constructed to detect patterns in the relative gene-expression dataset.

IBM SPSS Statistics for Windows software (IBM SPSS 27; IBM Corp., Armonk, NY, USA) was used to conduct the polynomial regression analysis, Pearson’s correlation analysis, and one- and two-way ANOVAs. The hierarchical clustering was executed using the package “ComplexHeatmap”, and PCA was executed using the “FactoMineR” package of the R software version 4.0.3 (R Core Team, 2020). Graphs were realized using Graph Pad Prism 8 (GraphPad, La Jolla, CA, USA). The results are presented as means with pooled SEM.

## 3. Results

### 3.1. Hatchability Performance

The hatchability seemed to be lower in the 1.5 AKG treatment group although the late embryonic mortality was also low in this group (Table 2). The highest live pipping chicks were seen in the 1.0 AKG treatment group, followed by the 1.5 AKG group. The chicks which pipped the eggs but died before hatching (dead pipping) were recorded as highest in the 1.5 AKG treatment group. A low number of dead pipping percentages was recorded in the U-CON and 1.0 AKG treatments. Finally, no dead pipping chicks were seen in the other two treatment groups. Hatchling weight and chick-weight-to-egg-weight ratio (CWEWR) were not significantly different between treatment groups (Table 2).

### 3.2. Organ Weights and Length

Table 3 presents the organ indices of chicks at hatching. A significant increase (*p* = 0.006) in the absolute and relative weights of the heart was seen in the 0.5 AKG group as compared to the 0 AKG group. The absolute (*p* = 0.039) and relative (*p* = 0.009) weights of the remnant yolk sac were found to quadratically increase according to the concentration of AKG. The absolute (*p* = 0.002) and relative (*p* = 0.008) liver weights of hatchlings were significantly higher in 0.5 AKG and 1.5 AKG groups as compared to 0 AKG. Further, a significant linear increase in the absolute (*p* = 0.040) and relative (*p* = 0.035) liver weights was found with the increasing concentration of AKG.

At 21 days of age, however, no significant differences were seen in all the treatment groups regarding absolute and relative weights of the liver, spleen, and bursa of Fabricius (Supplementary Table S1). No significant differences were seen among different treatment groups for the absolute and relative lengths of the small intestines and the average length of the cecum (Supplementary Table S2).

### 3.3. Growth Performances

The growth performances are represented in Table 4. A significant quadratic increase was observed for BW on day 7 (*p* = 0.051) and day 21 (*p* = 0.020) when increasing the AKG dose. Average daily gain (ADG) from days 8 to 21 also showed a significant quadratic increase for the first week (*p* = 0.057) and the next two weeks (*p* = 0.025). Average daily feed intake (ADFI) from days 8 to 21 was significantly (*p* = 0.048) lower in the 0.5 AKG group compared to all other treatments. In addition, ADFI (*p* = 0.036) from days 8 to 21 and feed-conversion ratio (FCR) (*p* = 0.028) from days 0 to 7 showed a quadratic increase with a higher concentration of in ovo AKG.

### 3.4. Plasma Metabolites

Table 5 represents the effects of in ovo feeding of AKG and age on plasma metabolites. Furthermore, 0.5 AKG significantly increased total protein concentrations (*p* = 0.025) as compared to 0 AKG, but the other parameters were not significantly unaffected. There was a significant age effect on plasma glucose, total protein, calcium, and cholesterol concentrations. Glucose (*p* < 0.001), total protein (*p* < 0.001), and calcium (*p* < 0.001) concentrations significantly increased with age, while cholesterol (*p* < 0.001) decreased.

### 3.5. Plasma and Hepatic Oxidative Stress Markers

Table 6 shows the effects of in ovo feeding of AKG, age, and their interactions on plasma oxidative stress markers. DPPH-RSA (%) decreased with age regardless of in ovo AKG (*p* < 0.001). In ovo AKG increased plasma DPPH-RSA (%) at day 0 (*p* < 0.001), but this effect disappeared at day 21, resulting in a significant interaction (*p* = 0.003) between age and AKG. For MDA, age, but not AKG (*p* = 0.08), was a decreasing factor (*p* < 0.001) with no interaction effect (*p* = 0.285). AB, the ratio of DPPH-RSA (%) to MDA, was significantly increased in a dose-dependent manner for AKG (*p* = 0.024). Although the balance increased significantly with age (*p* < 0.001), there was no interaction between the two (*p* = 0.503).

**Figure 1:**
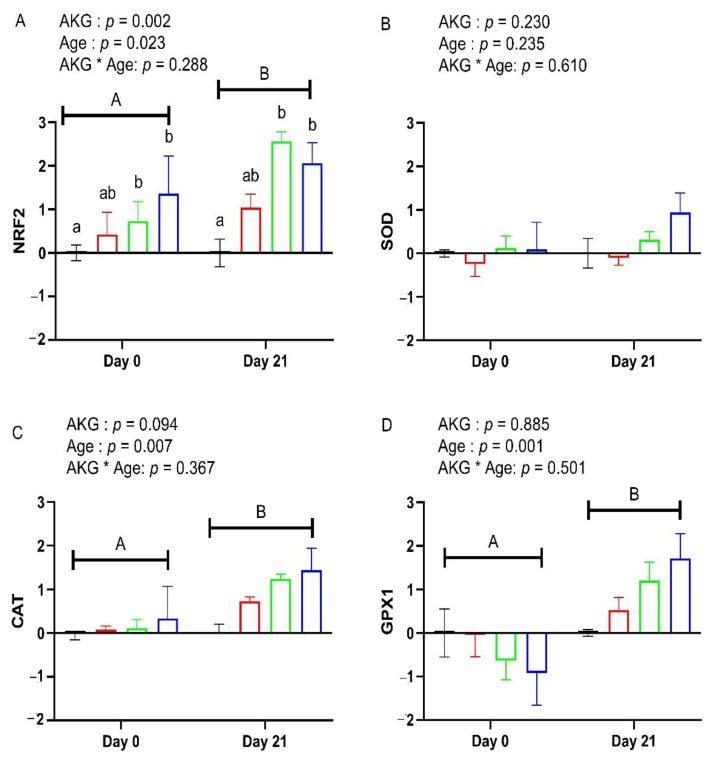
Effects of in ovo feeding of α-ketoglutaric acid (AKG) on hepatic mRNA expression of antioxidant-related genes NRF2 (**A**), SOD (**B**), CAT (**C**), GPX1 (**D**) at day 0 and day 21 of age in broilers. At 17.5 day of incubation, eggs were injected with 0.6 mL AKG solution with a concentration of 0% (0 AKG), 0.5% (0.5 AKG), 1.0% (1.0 AKG), and 1.5% (1.5 AKG) prepared in DDW, the chicks were raised in the thermoneutral environment as per standard guidelines of rearing. Data show mean ± SEM (n = 6). Means were analyzed by two-way ANOVA and Tukey post hoc test. A, B: means bearing different letters indicate a statistical difference (*p* < 0.05) at different ages of the broilers. a,b: means bearing different letters indicate a statistical difference (*p* < 0.05). Abbreviations: NRF2, nuclear factor erythroid 2-related factor; SOD, superoxide dismutase; CAT, catalase; GPX1, glutathione peroxidase 1.

Figure 1 indicates that AKG (*p* = 0.002) had a significant effect on the relative expression of the NRF2 gene. Furthermore, 1.0 AKG and 1.5 AKG treatments significantly upregulated NRF2 gene expression at hatching and day 21 in comparison to the 0 AKG. On the other side, a significant age effect was detected for NRF2 (*p* = 0.023), CAT (*p* = 0.007), and GPX1 (*p* = 0.001) at day 21 of age when compared with those at day 0.

**Figure 2:**
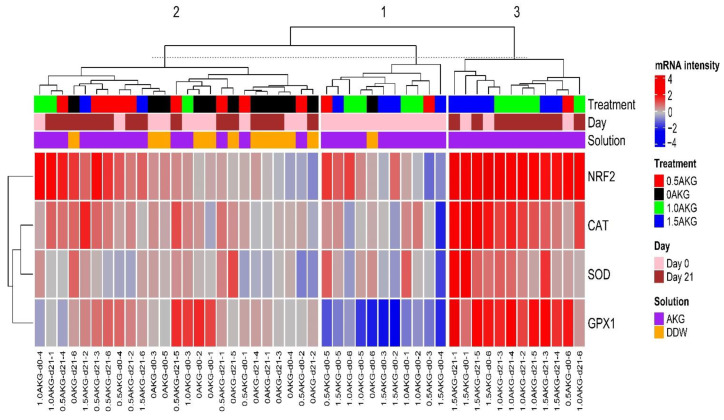
Heat map showing the hierarchical cluster of hepatic mRNA gene expression of antioxidant-related genes (NRF2, CAT, SOD, GPX1) at day 0 and day 21 old broilers. Each row represents a gene and each column represents an experimental unit belonging to a specific treatment. At 17.5 day of incubation, eggs were given an in ovo injection of 0%, 0.5%, 1.0%, and 1.5% AKG solution prepared in DDW, and the treatments are described as 0 AKG, 0.5 AKG, 1.0 AKG, and 1.5 AKG, respectively. The chicks were raised in a thermoneutral environment as per standard guidelines of rearing for the entire rearing period. RT-qPCR was used for gene expression analysis. GAPDH and β-actin were used as reference genes, the fold change (FC) of the genes was calculated as 2−ΔΔCt. The relative gene expression values were obtained as log2(FC). The tree was constructed using the package “ComplexHeatmap” of the R software version 4.0.3 (R Core Team, 2020). Abbreviations: NRF2, nuclear factor erythroid 2-related factor; CAT, catalase; SOD, superoxide dismutase; GPX1, glutathione peroxidase 1.

The gene regulation data were clustered vertically into three groups based on the Euclidian distances (Figure 2). The first cluster, located in the middle of the map, was composed of sampled hatchlings that mostly received AKG administration in ovo. These individuals presented a particularly strong downregulation (blue color) of GPX1 gene expression. The second cluster, which appears on the left side of the map, was much more heterogeneous and consisted of individuals exhibiting moderate regulation (light blue or bright red color) of all four antioxidant-related genes without particular distinction. Finally, a third cluster was located on the right side of the map. This cluster was dominated by birds (day 21) hatched from the eggs that received in ovo feeding of AKG. The birds of this cluster were characterized by the highest expression (red color) of all four genes evaluated during the trial.

**Figure 3:**
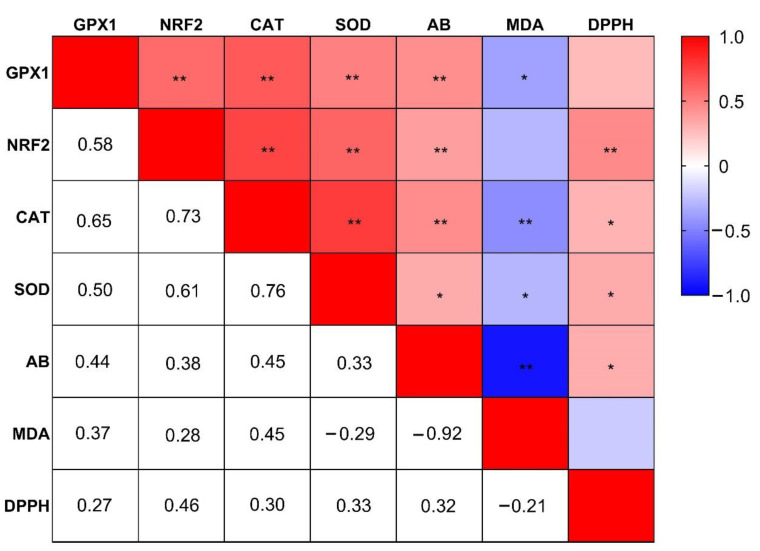
Pearson correlation heat map between hepatic antioxidant-related gene expressions and oxidative stress markers in plasma. The red color indicates a positive correlation; the blue color indicates a negative correlation and the white color indicates no correlation. Pearson r values were calculated using the IBM SPSS Statistics for Windows software (IBM SPSS 27; IBM Corp., Armonk, NY, USA). The heat map was realized using Graph Pad Prism 8 (GraphPad, La Jolla, CA, USA). Abbreviations: NRF2, nuclear factor erythroid 2-related factor; SOD, superoxide dismutase; CAT, catalase; GPX1, glutathione peroxidase 1; AB, antioxidant balance; MDA, malondialdehyde; DPPH, 2,2-diphenyl-1-picrylhydrazyl free radical scavenging activity. ** Correlation is statistically significant at the 0.01 level. * Correlation is statistically significant at the 0.05 level.

Figure 3 presents the Pearson’s correlation between the relative regulation of antioxidant-related genes in the liver, MDA concentration, DPPH-RSA (%), and AB in plasma. The overall mRNA expression of GPX1 was positively correlated (red) with the gene expression of NRF2 (r = 0.58, *p* < 0.01), CAT (r = 0.65, *p* < 0.01), SOD (r = 0.50, *p* < 0.01), AB (r = 0.44, *p* < 0.01), and negatively correlated (blue) with MDA concentration in plasma (r = 0.37, *p* < 0.05). Positive correlations were detected between NRF2 and CAT (r = 0.73, *p* < 0.01), NRF2 and SOD (r = 0.61, *p* < 0.01), NRF2 and AB (r = 0.38, *p* < 0.01), and NRF2 and DPPH-RSA (%) (r = 0.46, *p* < 0.01) in plasma. Further, the relative gene expression of CAT was positively related with SOD (r = 0.76, *p* < 0.01) in the liver, AB (r = 0.45, *p* < 0.01) and DPPH–RSA (%) (r = 0.30, *p* < 0.05), but negatively correlated with plasma MDA (r = 0.45, *p* < 0.01). Similarly, SOD was positively correlated with AB (r = 0.33, *p* < 0.05) and DPPH-RSA (%) (r = 0.33, *p* < 0.05), whereas it was negatively correlated with plasma MDA (r = −0.29, *p* < 0.05). A significant negative correlation was noticed between AB and MDA in plasma (r = −0.92, *p* < 0.01) whereas a positive correlation was seen between AB and DPPH-RSA (%) (r = 0.32, *p* < 0.05).

**Figure 4:**
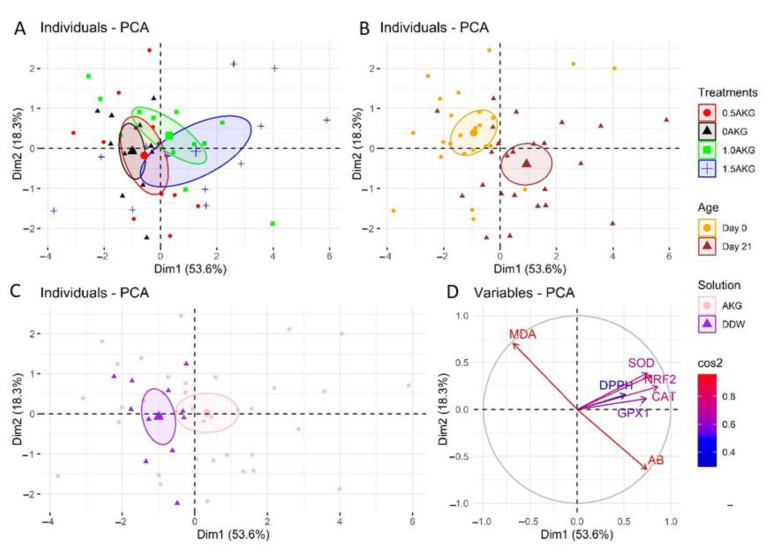
Principal component analysis (PCA) plot of individuals and variables. An individual refers to a sampled bird per treatment while a variable is a biological parameter analyzed. The individuals have been colored according to the treatments (**A**), age (**B**), and solution injected (**C**). The variables have been colored based on their squared cosine (**D**). The PCA was executed in R using the package “FactoMineR”. Abbreviations: NRF2, nuclear factor erythroid 2-related factor; SOD, superoxide dismutase; CAT, catalase; GPX1, glutathione peroxidase 1; AB, antioxidant balance; MDA, malondialdehyde; DPPH, 2,2-diphenyl-1-picrylhydrazyl free radical scavenging activity; Dim1, dimension 1; Dim2, dimension 2.

Figure 4 shows PCA plots obtained using antioxidant-related parameters. Individuals were projected on the first two dimensions of the PCA, which represented about 72% of the variability in the dataset. Additionally, to improve the contrast of the results, individuals were assigned different colors for each treatment (Figure 4A), the age of the bird at the time of sample collection (Figure 4B), and the injected solution (Figure 4C). Figure 4D shows that SOD, NRF2, and CAT were the variables most correlated with the first dimension, while MDA and AB were highly correlated with the second dimension. Figure 4B depicts a clear separation between clusters of birds sampled on day 0 (orange) and day 21 (brown) of age. As the orange cluster appears to be located higher and the brown cluster lower on the second dimension, it indicates that on day 0 birds exhibited higher plasma MDA concentration whereas, on day 21, birds tended to have higher AB (see also Table 6). Figure 4C shows distinct clusters of birds that received AKG (pink) and DDW (purple) as in ovo injection. The separation between these clusters was mainly located in the first dimension. Thus, the birds injected with AKG, located on the right side, showed higher overall gene expression of SOD, NRF2, and CAT. Antioxidant-associated genes were most strongly upregulated in chickens that received the highest concentration (1.5%) of AKG (mostly right-most) (Figure 4A).

## 4. Discussion

AKG has been identified as one of the most important speed determinants in the tricarboxylic acid cycle in most organisms [21]. To our knowledge, this is the first study to evaluate the effects of in ovo feeding of AKG in broilers. In tissues, AKG is readily converted to glutamine [23]. The in ovo injection of glutamine has not been shown to reduce the hatchability performances in meat-type breeder eggs [46]. We included the U-CON group in the study to determine the effect of in ovo methodology on the eggs/embryos. In the current study, we found the hatchability percentage of the 1.0AKG and 1.5 AKG groups to be numerically lower when compared to the other treatment groups. Indeed, this could be caused by a higher concentration of AKG. Similarly, another study using ornithine-AKG (OKG) reported a significantly lower hatchability in the treatment group injected with 1 mL 0.4% OKG [47]. However, the vehicle used in the above study was 0.75% physiological saline (1 mL) as compared to distilled water (0.6 mL) in our study. Differences in the solution volumes and osmolarity can be probable causes of changes in hatchability. Moreover, the chicks could hatch over a period of 24 to 48 h [48]. We noticed a higher proportion of live pipping chicks in the 1.0 AKG and 1.5 AKG groups, indicating a probability for the chicks to hatch a while after the completion of the 21-day incubation period. In addition, we did not notice any detrimental effect on BW and CWEWR (%) at hatching due to AKG injection, indicating that the in ovo feeding of AKG did not adversely affect these hatching parameters. Putting together, in ovo injection of AKG at low dose did not adversely affect hatching parameters.

The physiological functioning of cells in a living organism is greatly affected by the redox balance [49]. An imbalance in this equilibrium can lead to unwanted changes in the cells, including nucleic acid modification and cell death [49]. The birds at day 0 were presenting higher plasma MDA concentrations (Figure 4B). Due to an increase in oxygen utilization during the final stages of incubation, the ROS concentration increased [13]. These ROS may cause the oxidization of yolk lipids, increasing the MDA concentration on the day of hatching. DPPH-RSA (%) is a common way to estimate the TAC of plasma [50]. In our study, in ovo feeding of AKG increased TAC in plasma. Moreover, the AB increased linearly in a dose-dependent manner. AKG was shown as a potent anti-oxidative agent in vivo and in vitro in different methods [51]. AKG worked as a hepato-protective agent by participating in the non-enzymatic oxidative decarboxylation of hydrogen peroxide in rats with oxidative stress induced by ammonium acetate [32]. H_2_O_2_ concentrations decreased in various cells as AKG converted it to succinate [52]. Another mechanism is the conversion of AKG to glutathione via glutamate metabolism, which ultimately serves as a ROS scavenger [53]. AKG supplementation in aged mice helped improve the total antioxidant capacity [54]. Taken together, AKG acts as an antioxidant agent and can help scavenge the ROS produced by cells.

Oxidative damage is one of the main causes of liver damage [55]. Hence, to assess the effects of AKG as an antioxidative agent, we evaluated the mRNA expression of antioxidant-related enzyme genes in the liver. Glutathione peroxidase 1 (GPX1), catalase (CAT), and superoxide dismutase (SOD) are the primary antioxidant enzymes that remove oxidative intermediates formed in cells [56]. NRF2 is known as a master regulator of the enzymatic cascade responsible for alleviating the oxidative stress formed in cells [57]. Superoxide dismutase (SOD) is the spearhead enzyme participating in this process of ROS removal by scavenging the superoxide ion and converting it into hydrogen peroxide (H_2_O_2_) [58]. Glutathione peroxidase1 (GPX1) converts H_2_O_2_ into water (H_2_O) and helps create redox balance in the body [59]. Another enzyme, catalase (CAT) works in close propinquity with GPX1 [60]. As a result, the elimination of H_2_O_2_ is attained by the cooperative function of CAT and GPX1 [61]. In the current study, the expression of NRF2, SOD, CAT, and GPX1 was positively correlated with AB in plasma. Furthermore, the MDA concentration in plasma was found to be negatively correlated with the expression of GPX1, CAT, SOD, and AB in plasma. The activity of antioxidant enzymes (SOD and/or GPX) has also been shown to decrease with age in various rat models while oxidative damage increases [62,63 and 64]. We evaluated the expression of these genes at day 0 and day 21 of age. NRF2, CAT, and GPX1 were significantly upregulated at day 21, perhaps due to in ovo AKG feeding during the embryonic stage. NRF2 was significantly upregulated in the 1.0 AKG and 1.5 AKG groups. A higher expression of these genes at later stages of life could be an indication of higher antioxidant capacity. The effects of in ovo feeding can be manifested later in life, and include thermotolerance [65], improved meat quality [66], better antioxidation capacity [37], and increased expression of hepatic antioxidant genes [67]. The addition of 1% AKG in water helped mitigate ammonia-induced oxidative stress, with increased SOD, GPX, and CAT in the serum, gills, and intestine of hybrid sturgeon [67]. Hence, in the current study, the upregulation of these genes could also be imputed to in ovo AKG feeding, denoting that the birds would have a better antioxidant ability at later stages of life.

Furthermore, the heat map demonstrates three major clusters in the liver of broilers. Two clusters each show common characteristics, but a third one does not. One of the two clusters shows that in ovo AKG feeding moderately downregulated NRF2, CAT, SOD, and GPX1 at day 0. On contrary, the other cluster depicts that these genes were mostly upregulated at day 21, indicating that these genes were upregulated with age. AKG via glutamine production can alleviate ROS stress in cells. In the intestinal cells, glutamine can elevate glutathione concentrations [68,69]. Glutathione is one of the most potent ROS scavengers, reducing ROS during self-oxidation [70]. Moreover, glutamine can activate the NRF2/ARE signaling pathway and increase NRF2 gene expression [69,71]. SOD and CAT levels increased in murine liver due to the upregulation of NRF2 [72], conferring better protection against ROS damage. However, a direct role of AKG in NRF2 activation is yet to be established. In this study, we found that AKG helped improve plasma antioxidant capacity and notably upregulated hepatic mRNA expression of antioxidant-related genes. Thus, AKG can not only help in the non-enzymatic scavenging of ROS but also upregulates hepatic gene expression for enzymatic pathways. Future studies could provide better insights to demonstrate the direct effects of AKG as an antioxidant.

In broiler production, the consideration of organ weights is important. In our study, the 0.5 AKG and 1.5 AKG groups showed a significant increase in the weight of the liver. The liver is involved in a variety of bodily functions, ranging from the metabolism of nutrients and the storage of fat-soluble vitamins to detoxification, and as a site for phagocytosis of pathogens and dead RBCs through Kupffer cells [73]. AKG is known to be quickly metabolized by the hepatocytes to produce ATP [74]. Hence, AKG acts as a source of fuel energy for the hepatocytes. The role of the liver in glycogen storage is undeniable [75]. During the later stages of the incubation, the extent of gluconeogenesis is increased in the hepatocytes and the extra energy starts to be stored in the liver [76]. This energy is used for physical activities during hatching such as pipping by the chicks. An improvement in the liver yield could be an indicative indicator of a higher deposition of glycogen in the hepatocytes. This effect seems to increase with the concentration of AKG administered, as both absolute and relative liver weights linearly increased with the increase in AKG. Summing up, in ovo feeding of AKG aids in conferring a better liver index on day-old chicks, suggesting that during embryogenesis, a higher amount of energy is spared and stored in the form of glycogen. In this study, we did not analyze the glycogen content of the liver. This could be an interesting hypothesis to test in future studies.

Commercial broiler farms aim to achieve a higher output in poultry production. Although our treatments seem not to highly affect growth performances in broilers, we did find quadratic increases in BW, ADG, and ADFI during the grower phase. Moreover, dietary 1.0% AKG did not affect BW and daily FI in layers [26]. Moreover, oral supplementation of OKG did not affect BW in turkeys [24]. Whereas the dietary supplementation of 1.0% glutamine had a positive impact on BW, FCR, ADFI, ADG, and other production performances in broilers raised until 6 weeks of age [77], its effectiveness on BW has not been shown in others’ studies [78,79]. The discrepancy may be due to the species of birds, the mode of supplementation, and/or the compound employed. Although these studies were not exactly the same as the current study, the results of the current study fall in line with those of the previous studies [24,26,78,79]. Moreover, it is still unclear whether AKG’s effect on broiler performance is through glutamine metabolism. Together with the previous and current results, the in ovo feeding of AKG does not considerably affect production performances in broilers. Future studies with in ovo AKG feeding in broilers are important to reach further conclusions.

The analysis of various metabolites in plasma is considered an essential tool as it aids in identifying diseases and the metabolic status of the birds [80]. In ovo feeding of 0.5 AKG significantly increased the total plasma protein compared to 0 AKG. Comparable results were found in broilers fed 1% glutamine in the feed, resulting in an increase in total protein concentrations [77]. In addition, proline, glutamic acid, and leucine were reported as one of the most abundant amino acids in freeze-dried chicken-blood powder in a study comparing blood composition and functional properties of different species [81]. AKG helps in the anabolism of all the amino acids mentioned above. Dietary supplementation of AKG labeled with C^13^ in young piglets confirmed its role in the biosynthesis of certain amino acids such as glutamate, glutamine, leucine, and proline [82]. These results and the current study suggest the role of AKG in improving amino acid synthesis in broilers.

## 5. Conclusions

In summary, in ovo AKG injection did not seem detrimental to broiler embryos especially at the lower doses, but rather improved organ development in chicks at hatch. AKG improved the total antioxidant capacity of broilers, as evidenced by an increased DPPH-RSA (%), providing better protection against antioxidative damage to older birds. Furthermore, AKG also upregulated the hepatic expression of antioxidant-related genes (NRF2, SOD, CAT, and GPX1) mostly in the older birds, hence conferring better anti-oxidant status. Although there was no beneficial differential effect on the growth performance of broilers, the role of in ovo AKG as an antioxidant may be beneficial when exposed to adverse environmental conditions. The current study could serve as a preliminary study for further understanding the impact of in ovo feeding of AKG during stress and its impact on meat quality in broilers.

## Figures and Tables

**Table 1:** Oligonucleotide primer sequence for RT-qPCR.

S. No.	Gene	Sequence	Accession Number	Reference
1.	GAPDH	F: TTGGCATTGTGGAGGGTCTTA R: GTGGACGCTGGGATGATGTT	NM_204305.1	[43]
2.	β-actin	F: ACCGGACTGTTACCAACA R: GACTGCTGCTGACACCTT	NM_205518.1	[44]
3.	NRF2	F: CAGAAGCTTTCCCGTTCATAGA R: GACATTGGAGGGATGGCTTAT	NM_205117	[44]
4.	CAT	F: ACCAAGTACTGCAAGGCGAA R: TGAGGGTTCCTCTTCTGGCT	NM_001031215.1	[43]
5.	SOD	F: AGGGGGTCATCCACTTCC R: CCCATTTGTGTTGTCTCCAA	NM_205064.1	[43]
6.	GPX1	F: AACCAATTCGGGCACCAG R: CCGTTCACCTCGCACTTCTC	NM_001277853.2	[36]

Abbreviations: GAPDH, glyceraldehyde-3-phosphate dehydrogenase; β-actin, beta-actin; NRF2, nuclear factor erythroid 2-related factor; CAT, catalase; SOD, superoxide dismutase; GPX1, glutathione peroxidase 1.

**Table 2:** Effects of in ovo feeding of α-ketoglutaric acid (AKG) on hatchability parameters of broilers *.

Parameters	Treatments					Pooled SEM	*p*-Value		
	U-CON	0 AKG	0.5 AKG	1.0 AKG	1.5 AKG		ANOVA ^1^	Lin ^2^	Quad ^2^
Hatchability (%) #	88.71	85.48	88.89	80	79.31	NA	NA	NA	NA
Late EM (%) #	6.45	11.29	9.52	7.27	6.9	NA	NA	NA	NA
Live pipping (%) #	1.61	3.23	1.59	9.09	6.9	NA	NA	NA	NA
Dead pipping (%) #	3.23	0	0	3.64	6.9	NA	NA	NA	NA
Egg weight (g)	54.2	54.3	54.2	54.2	54.3	NA	NA	NA	NA
BW at hatch	36.8	36.8	36.7	36.6	36.9	0.121	0.978	0.961	0.274
CWEWR (%)	69.1	70.0	69.4	69.7	68.8	0.368	0.883	0.42	0.922

* At 17.5 day of incubation, eggs were treated with nothing (U-CON) or injected with 0.6 mL AKG solution with a concentration of 0% (0 AKG), 0.5% (0.5 AKG), 1.0% (1.0 AKG), and 1.5% (1.5 AKG). ^1^, *p* value of all the treatment groups. ^2^, *p* value of all the treatment groups except the U-CON. # All the values are calculated on fertile egg basis. Data are presented as mean (n = 6) and pooled SEM values. Abbreviations: EM, embryonic mortality; CWEWR, chick-weight-to-egg-weight ratio; Lin, linear effect; Quad, quadratic effect.

**Table 3:** Effects of in ovo feeding of α-ketoglutaric acid (AKG) on the absolute and relative organ weights of the gizzard, heart, liver, proventriculus, and yolk sac in chicks at hatching *.

Parameters	Treatments				Pooled SEM	*p*-Value		
	0 AKG	0.5 AKG	1.0 AKG	1.5 AKG		ANOVA	Lin	Quad
Absolute organ weight (g)								
Liver	0.70 ^a^	0.90 ^b^	0.80 ^ab^	0.88 ^b^	0.022	0.002	0.04	0.178
Yolk sac	4.47	3.68	3.49	4.38	0.172	0.091	0.767	0.039
Gizzard	1.68	1.86	1.82	1.82	0.047	0.609	0.377	0.377
Proventriculus	0.34	0.31	0.32	0.29	0.015	0.687	0.331	0.917
Heart	0.22 ^a^	0.29 ^b^	0.23 ^a^	0.27 ^ab^	0.008	0.006	0.32	0.436
Relative organ weight (g/g BW)								
Liver	1.97 ^a^	2.47 ^b^	2.24 ^ab^	2.43 ^b^	0.062	0.008	0.035	0.185
Yolk sac	12.52	10.06	9.83	12.07	0.461	0.072	0.715	0.009
Gizzard	4.72	5.09	5.14	5.04	0.136	0.716	0.414	0.401
Proventriculus	0.94	0.84	0.91	0.79	0.043	0.582	0.316	0.914
Heart	0.62 ^a^	0.79 ^b^	0.64 ^a^	0.74 ^ab^	0.021	0.006	0.277	0.387

* At 17.5 day of incubation, eggs were injected with 0.6 mL AKG solution with a concentration of 0% (0 AKG), 0.5% (0.5 AKG), 1.0% (1.0 AKG), and 1.5% (1.5 AKG). Chicks obtained from each treatment group at hatching were sampled and the different organ weights were recorded. Data are presented as mean ± SEM (n = 6). ^a,b^: means bearing different letters differ significantly in the same row (*p* < 0.05). Abbreviations: BW, body weight; Lin, linear effect; Quad, quadratic effect.

**Table 4:** Effects of in ovo feeding of α-ketoglutaric acid (AKG) on growth parameters of broilers from day 0 to day 21 of rearing *.

Parameters	Treatments				Pooled SEM	*p*-Value		
	0 AKG	0.5 AKG	1.0 AKG	1.5 AKG		ANOVA	Lin	Quad
BW (g/bird)								
Day 0	36.8	36.7	36.6	36.9	0.073	0.669	0.961	0.274
Day 7	144.2	134.0	139.0	144.5	2.000	0.199	0.744	0.051
Day 21	836.4	764.3	795.3	838.4	12.525	0.100	0.749	0.020
ADG (g/bird)								
0 to 7 days	15.3	13.9	14.6	15.4	0.290	0.218	0.746	0.057
8 to 21 days	49.5	45.0	46.9	49.6	0.801	0.123	0.761	0.025
ADFI (g/bird)								
0 to 7 days	15.4	14.5	15.1	15.5	0.264	0.554	0.696	0.228
8 to 21 days	67.5 ^ab^	63.6 ^a^	67.2 ^ab^	70.3 ^b^	0.877	0.048	0.125	0.036
FCR								
0 to 7 days	1.00	1.05	1.03	1.01	0.008	0.148	0.924	0.028
8 to 21 days	1.37	1.42	1.44	1.42	0.012	0.189	0.084	0.167

* At 17.5 day of incubation, eggs were injected with 0.6 mL AKG solution with a concentration of 0% (0 AKG), 0.5% (0.5 AKG), 1.0% (1.0 AKG), and 1.5% (1.5 AKG). Chicks obtained from each treatment group at hatching were reared as per standard temperature and humidity guidelines. Data are presented as mean (n = 6) and pooled SEM values. ^a,b^: means bearing different letters differ significantly in the same row (*p* < 0.05). Abbreviations: ADG, average daily gain; ADFI, average daily feed intake; BW, body weight; FCR, feed-conversion ratio; Lin, linear effect; Quad, quadratic effect.

**Table 5:** Effects of in ovo feeding of α-ketoglutaric acid (AKG) on glucose, total protein, triglyceride, cholesterol, and calcium concentrations in plasma on day 0 and day 21 of age *.

Treatments		Glucose	Total Protein	Triglycerides	Cholesterol	Calcium
AKG effect						
0 AKG		260.8 ± 14.4	2.5 ± 0.2 ^a^	21.2 ± 6.1	261.8 ± 37.8	10.1 ± 0.4
0.5 AKG		252.3 ± 8.9	2.9 ± 0.2 ^b^	57.8 ± 4.4	255.8 ± 37.4	10.6 ± 0.3
1.0 AKG		254.3 ± 13.3	2.8 ± 0.2 ^ab^	43.3 ± 5.4	248.0 ± 38.6	10.9 ± 0.3
1.5 AKG		248.7 ± 12.3	2.8 ± 0.2 ^ab^	42.8 ± 4.1	238.2 ± 32.5	10.4 ± 0.3
*p*-value		0.073	0.025	0.124	0.493	0.066
Day effect						
Day 0		223.6 ± 4.1	2.2 ± 0.1	51.0 ± 2.7	366.8 ± 10.7	9.7 ± 0.2
Day 21		284.3 ± 7.2	3.3 ± 0.1	43.2 ± 4.4	135.0 ± 3.2	11.3 ± 0.2
*p*-value		0.001	0.001	0.130	0.001	0.001
AKG * Day effect						
Day 0	0 AKG	232.7 ± 13.4	1.9 ± 0.1	50.3 ± 4.9	381.5 ± 22.2	8.9 ± 0.2
	0.5 AKG	227.0 ± 2.6	2.3 ± 0.1	55.3 ± 6.4	375.8 ± 19.2	9.9 ± 0.2
	1.0 AKG	222.0 ± 4.6	2.2 ± 0.1	49.7 ± 6.6	370.2 ± 23.5	10.2 ± 0.4
	1.5 AKG	213.0 ± 8.0	2.3 ± 0.1	48.7 ± 5.0	339.8 ± 21.9	9.7 ± 0.2
Day 21	0 AKG	289.0 ± 20.5	3.0 ± 0.1	38.7 ± 11.3	142.0 ± 7.2	11.3 ± 0.4
	0.5 AKG	277.5 ± 9.3	3.4 ± 0.2	60.3 ± 6.4	135.8 ± 6.2	11.3 ± 0.2
	1.0 AKG	286.5 ± 18.5	3.4 ± 0.2	36.8 ± 8.3	125.8 ± 6.7	11.5 ± 0.4
	1.5 AKG	284.3 ± 9.5	3.3 ± 0.2	37.0 ± 6.1	136.5 ± 4.7	11.2 ± 0.2
*p*-value		0.842	0.816	0.546	0.550	0.230

* At 17.5 day of incubation, eggs were injected with 0.6 mL AKG solution with a concentration of 0% (0 AKG), 0.5% (0.5 AKG), 1.0% (1.0 AKG), and 1.5% (1.5 AKG). Blood was collected from chicks in each treatment group. Plasma was separated. Plasma was used to analyze the concentration of different metabolites. Data show mean ± SEM (n = 6). Means were analyzed by two-way ANOVA and Tukey post hoc test. ^a,b^: Means bearing different letters indicate a statistical difference (*p* < 0.05). Abbreviations: Lin, linear effect; Quad, quadratic effect.

**Table 6:** Effects of in ovo feeding of α-ketoglutaric acid (AKG) on the plasma antioxidant capacity indicated as DPPH-RSA (%), MDA concentration and antioxidant balance (AB) on day 0 and day 21 of age *.

Treatments		DPPH-RSA (%)	MDA (nmol/mL)	Antioxidant Balance (U)
AKG effect				
0 AKG		27.3 ± 1.0 ^a^	1.2 ± 0.1	21.4 ± 1.5
0.5 AKG		28.4 ± 1.4 ^b^	1.1 ± 0.1	23.6 ± 2.4
1.0 AKG		28.3 ± 1.2 ^b^	1.1 ± 0.1	24.7 ± 3.0
1.5 AKG		28.8 ± 1.1 ^b^	0.9 ± 0.1	29.0 ± 2.1
*p*-value	ANOVA	0.001	0.080	0.053
	Linear	0.381	0.066	0.024
	Quadratic	0.797	0.390	0.644
Day effect				
Day 0		32.1 ± 0.3	1.3 ± 0.1	20.2 ± 1.0
Day 21		24.3 ± 0.2	0.9 ± 0.1	29.2 ± 1.7
*p*-value		0.001	0.001	0.001
AKG * Day effect				
Day 0	0 AKG	30.5 ± 0.6 ^y^	1.2 ± 0.1	19.4 ± 0.9
	0.5 AKG	33.1 ± 0.3 ^z^	1.4 ± 0.1	18.1 ± 2.2
	1.0 AKG	32.2 ± 0.5 ^z^	1.4 ± 0.2	19.5 ± 2.6
	1.5 AKG	32.6 ± 0.1 ^z^	1.1 ± 0.1	23.6 ± 1.6
Day 21	0 AKG	24.0 ± 0.2 ^x^	1.1 ± 0.1	23.4 ± 2.7
	0.5 AKG	23.7 ± 0.4 ^x^	0.9 ± 0.1	29.1 ± 2.9
	1.0 AKG	24.5 ± 0.1 ^x^	0.9 ± 0.1	29.9 ± 4.7
	1.5 AKG	25.1 ± 0.3 ^x^	0.7 ± 0.1	34.4 ± 2.2
*p*-value		0.003	0.285	0.503

* At 17.5 day of incubation, eggs were injected with 0.6 mL AKG solution with a concentration of 0% (0 AKG), 0.5% (0.5 AKG), 1.0% (1.0 AKG), and 1.5% (1.5 AKG). Blood was collected from chicks in each treatment group. Plasma was separated. Plasma was used to analyze the different antioxidative markers. Antioxidant balance (AB) was derived as the ratio of DPPH-RSA to MDA in plasma. Data show mean ± SEM (n = 6). Means were analyzed by two-way ANOVA and Tukey post hoc test. ^a,b^: means bearing different letters indicate a statistical difference (*p* < 0.05). ^x, y, z^: means bearing different superscript indicate a statistical difference (*p* < 0.05) in interaction of AKG*Age. Abbreviations: DPPH-RSA (%), 2,2-diphenyl-1-picrylhydrazyl free radical scavenging activity; MDA, malondialdehyde.

## Data Availability

The data for this study is contained within the article.

## Supplementary Materials

The following supporting information can be downloaded at: https://www.mdpi.com/article/10.3390/antiox11112102/s1. Table S1: Effect of in ovo administration of AKG on the relative and absolute organ weight of liver, spleen and bursa of Fabricius of broilers at day 21 of age *, Table S2: Effects of in ovo feeding of α-ketoglutaric acid (AKG) on the absolute and relative length of different parts of the small intestine and average caecum length of broilers at day 21 of age *.
