# Supplementary material for: Effects of In Ovo Injection of α-Ketoglutaric Acid on Hatchability, Growth, Plasma Metabolites, and Antioxidant Status of Broilers
Source: Antioxidants (Basel). 2022 Oct 25;11(11):2102. doi: 10.3390/antiox11112102 (PMC9686527; doi:10.3390/antiox11112102)
Supplement: Supplementary file 1 [file antioxidants-11-02102-s001.zip › antioxidants-1985161-supplementary.pdf]

Table S1. Effect of *in ovo* administration of AKG on the relative and absolute organ weight of liver, spleen and bursa of Fabricius of broilers at day 21 of age*.

Parameters	Treatments				Pooled SEM	p-value		
	0AKG	0.5AKG	1.0AKG	1.5AKG		ANOVA	Lin	Quad
**Absolute organ weight (g)**								
Liver	27.38	28.09	28.17	28.00	0.568	0.965	0.712	0.713
Spleen	0.73	1.12	0.72	0.74	0.063	0.054	0.533	0.146
Bursa of Fabricius	1.49	1.39	1.37	1.36	0.060	0.891	0.472	0.724
Relative organ weight (g/ 100g BW)								
Liver	3.37	3.72	3.69	3.45	0.085	0.39	0.791	0.09
Spleen	0.09	0.15	0.10	0.09	0.010	0.071	0.584	0.096
Bursa of Fabricius	0.18	0.18	0.18	0.17	0.006	0.359	0.329	0.682

*At 17.5 day of incubation, eggs were injected with 0.6mL AKG solution with a concentration of 0% (0AKG), 0.5% (0.5AKG), 1.0% (1.0AKG), and 1.5% (1.5AKG). Chicks obtained from each treatment group at hatch were reared as per standard temperature and humidity guidelines. At day 21 of age, chickens from each treatment groups were sampled and the different organ weights were recorded. Data are presented as Mean ± SEM (n=6). Abbreviations: Lin, linear effect; Quad, quadratic effect.

Table S2. Effects of *in ovo* feeding of α-ketoglutaric acid (AKG) on the absolute and relative length of different parts of the small intestine and average caecum length of broilers at day 21 of age*.

Parameters	Treatments				Pooled	p-value		
	0AKG	0.5AKG	1.0AKG	1.5AKG	SEM	ANOVA	Lin	Quad
**Absolute length (cm)**								
Duodenum	25.67	25.50	23.83	25.00	0.381	0.330	0.292	0.392
Jejunum	57.67	57.50	53.17	57.67	1.120	0.420	0.675	0.317
Ileum	53.50	53.33	53.83	56.67	0.809	0.443	0.172	0.356
Relative length (cm/100g BW)								
Duodenum	3.17	3.37	3.12	3.10	0.068	0.491	0.464	0.425
Jejunum	7.10	7.64	7.05	7.09	0.199	0.713	0.734	0.552
Ileum	6.59	7.02	7.12	7.07	0.180	0.742	0.355	0.518
Cecum#	14.46	13.71	13.21	14.21	0.238	0.264	0.569	0.069

***At 17.5** day of incubation, eggs were injected with 0.6mL AKG solution with a concentration of 0% (0AKG), 0.5% (0.5AKG), 1.0% (1.0AKG), and 1.5% (1.5AKG). Chicks obtained from each treatment group at hatch were reared as per standard temperature and humidity guidelines. At day 21 of age, chickens from each treatment groups were sampled and the different organ lengths were recorded. Data are presented as Mean ± SEM (n=6). Abbreviations: Lin, linear effect; Quad, quadratic effect.

Cecal length was calculated as an average of both sides of the ceca.
